# Application of Baculovirus Expression Vector system (BEV) for COVID-19 diagnostics and therapeutics: a review

**DOI:** 10.1186/s43141-022-00368-7

**Published:** 2022-07-06

**Authors:** Muhammad Azharuddin Azali, Salmah Mohamed, Azian Harun, Faezahtul Arbaeyah Hussain, Shaharum Shamsuddin, Muhammad Farid Johan

**Affiliations:** 1 Department of Haematology, School of Medical Sciences, Universiti Sains Malaysia, 16150 Kubang Kerian, Kelantan Malaysia; 2 School of Agriculture Science and Biotechnology, Faculty of Bioresources and Food Industry, Universiti Sultan Zainal Abidin, 22200 Besut, Terengganu Malaysia; 3 Department of Medical Microbiology and Parasitology, School of Medical Sciences, Universiti Sains Malaysia, 16150 Kubang Kerian, Kelantan Malaysia; 4 Department of Pathology, School of Medical Sciences, Universiti Sains Malaysia, 16150 Kubang Kerian, Kelantan Malaysia; 5 School of Health Sciences, Universiti Sains Malaysia, 16150 Kubang Kerian, Kelantan Malaysia

**Keywords:** SARS-CoV-2, COVID-19, Baculovirus expression system, Spike protein, Non-structural protein, Angiotensin converting enzyme 2, Recombinant antibodies, Therapeutic proteins

## Abstract

**Background:**

The baculovirus expression vector system has been developed for expressing a wide range of proteins, including enzymes, glycoproteins, recombinant viruses, and vaccines. The availability of the SARS-CoV-2 genome sequence has enabled the synthesis of SARS-CoV2 proteins in a baculovirus-insect cell platform for various applications.

**Main body of the abstract:**

The most cloned SARS-CoV-2 protein is the spike protein, which plays a critical role in SARS-CoV-2 infection. It is available in its whole length or as subunits like S1 or the receptor-binding domain (RBD). Non-structural proteins (Nsps), another recombinant SARS-CoV-2 protein generated by the baculovirus expression vector system (BEV), are used in the identification of new medications or the repurposing of existing therapies for the treatment of COVID-19. Non-SARS-CoV-2 proteins generated by BEV for SARS-CoV-2 diagnosis or treatment include moloney murine leukemia virus reverse transcriptase (MMLVRT), angiotensin converting enzyme 2 (ACE2), therapeutic proteins, and recombinant antibodies. The recombinant proteins were modified to boost the yield or to stabilize the protein.

**Conclusion:**

This review covers the wide application of the recombinant protein produced using the baculovirus expression technology for COVID-19 research. A lot of improvements have been made to produce functional proteins with high yields. However, there is still room for improvement and there are parts of this field of research that have not been investigated yet.

**Supplementary Information:**

The online version contains supplementary material available at 10.1186/s43141-022-00368-7.

## Background

The baculovirus expression vector system (BEV) has been in use for more than 30 years [1]. Thousands of proteins have been successfully created using the BEV platform, and it is no longer limited to research purposes; it is now being used on a broad scale to manufacture a variety of biological products, including vaccines. At first, only two BEV-derived vaccines were commercialized [2]. Since then, numerous products have been approved for usage, including the latest Novavax’s COVID-19 vaccine [3]. BEV is made up of three parts: a transfer plasmid with a foreign gene to be inserted into the baculovirus genome, a baculovirus vector (i.e., bacmid) or baculovirus DNA (linearized DNA), and insect cell lines [4]. Strong promoters, such as the polyhedrin promoter, govern the transcription of the gene insert [5]. The original polyhedrin gene is replaced with multiple cloning sites for the insertion of the foreign gene downstream of the promoter [6]. The plasmid is then co-transfected with baculovirus DNA into insect cells [7]. Homologous recombination will be used to integrate the recombinant gene into the baculovirus genome [8]. Large-scale protein expression has been modified to incorporate larvae such as silkworm larvae. Using larvae instead of cells to make more protein has a number of benefits, such as increasing capacity and lowering the cost of making protein on a large scale [9].

The BEV platform has been used to express a wide range of proteins, including enzymes, glycoproteins, recombinant viruses, and vaccines [10]. The application of baculovirus as a vector for DNA delivery in humans is relatively safe because the virus cannot replicate in humans [11]. Moreover, the BEV has various advantages compared to other expression systems. The production of protein in the BEV is relatively simple compared to the mammalian expression system, which requires a CO_2_ incubator. However, unlike bacteria, it still has the post-translational capabilities to produce the glycosylated protein. In fact, baculovirus has been engineered to introduce a complex glycan structure to the recombinant protein that is normally found in mammals [12]. Moreover, the yield of the recombinant protein produced in BEV can be increased further using the baculovirus surface display method, where the ORF of the gene is fused in between the signal peptide of the BV glycoprotein GP64 and the mature GP64 sequence, causing the protein to fold correctly and become immunogenic enough to be taken by antigen-presenting cells [13]. The application of nanotechnology has sped up vaccine development, which includes the first mRNA vaccines for COVID-19 [14]. The nanoparticles act as carriers to protect the vaccine and get it to the target site.

As the number of COVID-19 patients increases around the world, scientists are focusing their efforts on every aspect of the disease, from diagnostics to therapies, by leveraging new technologies or exploiting existing platforms like BEV. This review gathers all the information from major databases about the application of BEV for the development of COVID-19 diagnosis and treatments to give insight about the achievement and limitations as well as opportunities in this field. This article also reviews the integration of nanotechnology into COVID-19 diagnostic and therapeutic research.

**Fig. 1:**
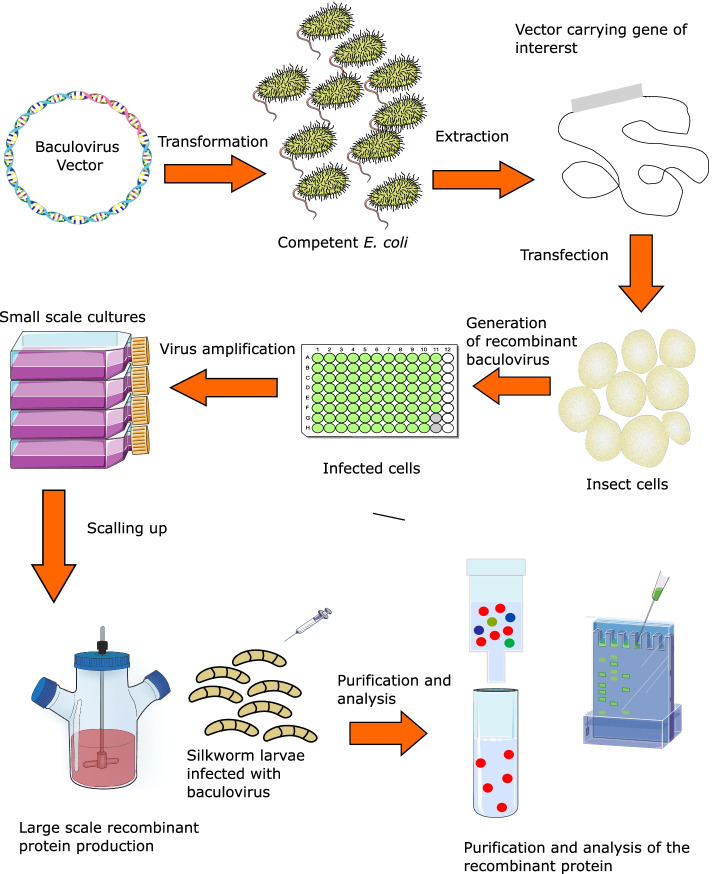
An overview of the general process for SAR-CoV-2 protein production in BEV. A baculovirus vector encoding the SARS-CoV-2 protein is transformed into competent *E. coli*. Translocation occurred in *E. coli* where the gene encoding SARS-CoV-2 protein was transferred from the donor plasmid to the bacmid. The bacmid is extracted from *E. coli* and used to transfect insect cells for the generation of recombinant baculoviruses. The recombinant baculovirus is further amplified for up scaling of the recombinant protein production. High-scale protein production is produced in bioreactors or by silkworm larvae. The protein is purified and analyzed before being used for various applications

The general process of producing SARS-CoV-2 proteins in BEV is shown in Fig. 1.

## Literature survey

**Fig. 2:**
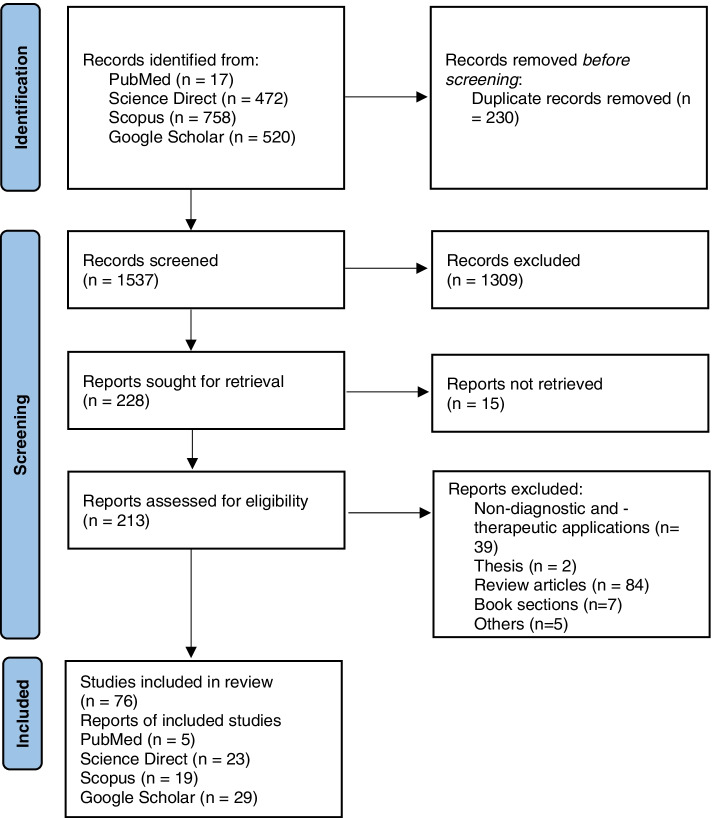
PRISMA chart. The flow chart shows a detailed presentation of the number of studies retrieved, deduplicated, excluded during screening, and included in this review

Literature resources were searched from PubMed, ScienceDirect, Google Scholar, and Scopus databases using keyword combinations such as baculovirus expression AND (sars-cov-2 OR 2019nCoV OR severe acute respiratory syndrome coronavirus 2). The specific queries for each database are given in Table S1. The inclusion criteria were any study or article related to the production of SARS-CoV-2 protein using baculovirus expression systems. Articles that reported the use of expression systems other than baculovirus or the production of recombinant protein from viruses other than SARS-CoV-2 were excluded. Only publications in English were selected. In addition, only original articles reporting the production of SARS-CoV-2 proteins for diagnostic and therapeutic research were chosen. The Preferred Reporting Items for Systematic Reviews and Meta-Analyses (PRISMA) chart for this review is shown in Fig. 2.

## The SARS-CoV-2 proteins produced in BEV

SARS-CoV-2 viral particles consist of spikes (S), membranes (M), envelopes (E), and nucleocapsid (N) proteins [15]. The S protein binds to the human angiotensin converting enzyme 2 (hACE2) via its receptor-binding domain (RBD) during SARS-CoV-2 infection [16]. The S and N proteins have been discovered to be viral materials that can be utilized to detect coronavirus infections, such as SARS-CoV-2 [17]. The signal peptide, the S1 subunit, and the S2 subunit make up the spike protein [18]. The S1 subunit consists of an N-terminal domain (NTD), and a C-terminal domain (CTD) where the RBD is located [19].

A single purification yielded both non-glycosylated and glycosylated forms of the S protein, as well as other insect cell proteins [20]. In insect cells, the glycosylated S protein is decorated with 38 *N*-glycans, which mostly consist of oligomannose (Hex) and fucose (Fuc), while in human cells, the S protein is glycosylated with 157 N-glycans, mainly containing extra N-acetylglucosamine (HexNAc) and galactose (Hex), variably terminating with sialic acid (NeuAc) [21]. Glycosylation at the binding site and the proximate amino acid can affect the interaction between the spike protein and the host cell [22]. It was reported that the glycosylated S1 showed lower binding affinities to ACE2 as compared to the non-glycosylated [23]. Additionally, molecular dynamic (MD) simulations show that glycosylation creates steric effects that have an impact on the interaction between the N-terminal sequence of S1 and ACE2. However, the low molecular size of the *N*-glycans synthesized in insect cells produces lower steric effects as compared to mammalian cells. The Coulombic repulsion, which drives S1 away from ACE2, is absent in proteins derived from insect cells. This is because sialylation occurs only in *N*-glycans that are synthesized by mammalian cells.

**Fig. 3:**
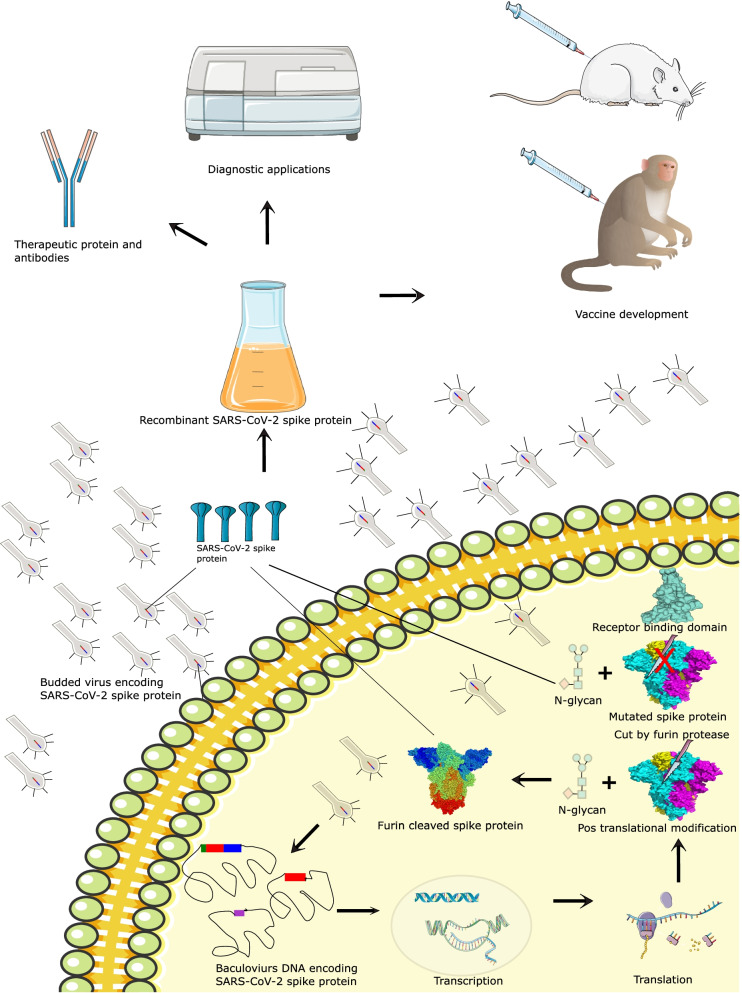
The production of SAR-CoV-2 spike protein or its subunits in BEV. Infected insect cells produce wild-type or mutated spike protein, or RBD. The wild-type spike protein is cleaved by furin protease but not the mutated protein. All the proteins produced by the insect cell will be glycosylated. The recombinant proteins are used for diagnostic and therapeutic studies such as vaccines, therapeutic proteins, and antibody development

The furin protease produced by insect cells breaks the synthesized S protein into S1 and S2 subunits [16]. Besides, recombinant SARS-CoV-2 proteins produced in silkworm larvae were also exposed to furin protease digestion [24]. The in-cell digestion resulted in the loss of the N-terminal signal peptide from the C-terminal half of S protein, preventing the truncated form of the protein from being secreted to silkworm serum. As a result, less protein could be recovered. The removal of a furin-recognized polybasic cleavage site and the inclusion of a stabilizing mutation in the S2 subunit by replacing K986P and V987P with prolines can stabilize and improve S protein production (Fig. 3) [16]. This modification keeps the S protein from extending its central helix, which keeps it in its prefusion state [25].

## Application of baculovirus expression vector system (BEV) for SARS-CoV-2 diagnostics and therapeutics

### Diagnostic applications

The widespread transmission of SARS-CoV-2 necessitates a quick and accurate diagnosis of COVID-19 disease. COVID-19 is diagnosed using either molecular or serum-specific antibody detection assays [26]. Antibody detection against SARS-CoV-2 is beneficial for analyzing infected patients' immune responses, determining the rate of infection in specific areas, and identifying a person with strong antibody responses who might be a donor for convalescent serum or plasma therapies [27]. The two assays that have been approved for the diagnosis of COVID-19 are the enzyme-linked immunosorbent assay (ELISA) and lateral flow [17]. The BEV-derived spike protein has been used to diagnose COVID-19 disease (Table 1) [27].

**Table 1:** Recombinant proteins produced at laboratory scale using BEV for diagnosis of COVID-19

**Proteins**	**Modifications**	**Host Cells**	**Purification Method**	**Yield**	**Purity **	**Specific Applications**	**References**
Moloney MurineLeukemia Virus Reverse Transcriptase (MMLV-RT)	TEV-8xHis-Strep tag added	Sf9	Ni-NTA affinity chromatography	7.5 mg/l	95%	qRT-PCR	[15]
Spike Protein, S1 and receptor binding domain.	Signal peptides, T4 foldon trimerization domain, cleavage sites and peptide tags added. Mutations introduced to the sequences.	Sf9/High Five/*Tnao*38/*Tnms*42/*Rachiplusia nu* larvae	Ni-NTA affinity chromatography	0.5 – 1.5 mg/l or 15 ug/g larvae	Up to 99%	ELISA	[27–31]
Spike and nucleocapsid protein	Removal of transmembrane and endodomains. Kozak motif, IZN4 fold on trimerization, thrombin cleavage site and hexahistidine tag added.	Sf9/High Five	Ni-NTA affinity chromatography	Not measured	Not measured	mPLEX-CoV assay	32]

The spike protein was used to coat 96-well plates in ELISA for the detection of IgG, IgA, or IgM antibodies in the positive serum, with IgG being the most preferred antibody because it is upregulated 1–7 days after COVID-19 detection and its level remains high and detectable for at least 3 months [33]. In an ELISA test, it was discovered that COVID-19 sera responded highly to full-length S protein compared to RBD [27]. Furthermore, the test was able to distinguish between serum from COVID-19 patients and serum from convalescent samples. Most notably, the test was highly specific such that it does not detect other human viruses’ infections, including the human immunodeficiency virus (HIV) and other human coronaviruses. The use of S protein for anti-SARS-CoV-2 antibody detection was further expanded with fluorometric detection methods [29]. Human serum spiked with monoclonal IgM anti-COVID-19 was used to evaluate a microcapillary film (MCF) containing an array of 10 micro-capillaries coated with 15 μg/ml SARS-CoV2-S1. The strip was coated with the AttoPhos® AP Fluorescent Substrate System (Promega). Then the secondary antibody anti-human IgG or IgM conjugated with alkaline phosphatase was added to generate the fluorescent signal. The signal was captured with a camera and analyzed with ImageJ software.

The BEV platform was also used to synthesize subdomains of SARS-CoV-2 S proteins, such as S1 and its subdomain, RBD, instead of full-length spike proteins, where in some cases, the S1 protein was found to be the best performing antigen, such as in a Luminex-based suspension immunoassay (SIA) [34]. The S1 subunit was efficiently secreted with the addition of a signal peptide to the gene construct, and the S1 subunit was detectable in both cell extracts and supernatant, as opposed to the full-length spike protein [30]. The proteins were generated in large quantities in *Tnao*38 cells, and S1 protein purification in the supernatant yielded an 85% pure protein with a yield of 1 mg/l (Table 1). Tnao38 and High Five cells produce significantly more recombinant protein than Sf9 cells, although Sf9 cells have a higher capacity for producing infectious virus particles [35]. As a result, Sf9 cells are employed to make recombinant baculovirus while *Tnao*38 or High Five cells are used for large-scale protein production. For low-cost production, the large-scale SARS-CoV-2 spike protein is also produced in insect larvae such as silkworms and *Rachiplusia nu* [28]. A commercial ELISA test has been developed from RBD antigen which can be performed manually using basic laboratory equipment [31]. Its performance was no different from that of automatic ELISA if a high-quality antigen was used.

In other studies, a SARS-CoV-2 nucleocapsid protein was produced in BEV using a suspension culture of insect cells [36]. A total of 1 mg of protein was purified from 50 mg of crude protein. S and N proteins coupled to magnetic beads were used for the detection of anti-SARS-CoV-2 antibodies in the serum samples [32]. Apart from producing SARS-CoV-2 protein, baculovirus is also used for generating SARS-CoV-2 pseudovirus that can be handled in BSL-2 laboratories, which are more widely available compared to BSL-3 containment [37]. A SARS-CoV-2 spike protein pseudotyped baculovirus was created for the infection model study. It was detected using an anti-spike antibody attached to gold nanoparticles.

Non-SARS-CoV-2 proteins, such as Moloney murine leukemia virus reverse transcriptase (MMLVRT), were also produced in BEV for use in the one-step reverse-transcription quantitative polymerase chain reaction (qRT-PCR) for the diagnosis of COVID-19 [15]. The protein was synthesized in suspension culture and has a high concentration as well as purity (Table 1). The MMLVRT's integration into RT-PCR allowed for the detection of as few as 10 copies of RNA. The results were comparable with those from commercial RT-PCR kits.

So far, only spike and nucleocapsid proteins have been produced in BEV for the diagnosis of COVID-19. The membrane protein of SARS-CoV-2 has been produced in other expression systems such as the *E. coli* expression system [38]. Hence, the potential of the M protein to be produced in BEV for diagnostic purposes remains to be explored further, especially with the emerging of new variants that reduce the sensitivity of the current diagnosis of COVID-19 disease [39]. In addition, there is an urgent need for rapid and simple tests that can detect a wide range of SARS-CoV-2 variants.

Nanoparticle-based biosensors that provide high sensitivity and fast diagnosis are gaining interest [40]. A nano-biosensor for SARS-CoV-2 spike protein detection was developed by coating graphene sheets of the field-effect transistor (FET) with a specific antibody against SARS-CoV-2 spike protein [41]. Other applications of nanotechnology for detection of SARS-CoV-2 spike protein include surface engineering of mixed SAMs of CH_3_ and COOH groups coupled with anti-spike glycoprotein, membrane-engineered Vero cells, and fluorine tin oxide (FTO) electrode with gold nanoparticle (AuNPs) [42, 43]. Nanomaterials used for detecting SARS-CoV-2 nucleic acids include gold nanoparticles and poly (amino ester) carboxyl groups (PC)-coated magnetic nanoparticles (pcMNPs) [44–47]. AuNPs are also used for the detection of SARS-CoV-2 IgG-IgM combined antibodies [48]. The RdRP coding sequences were used to detect the SARS-CoV-2 infection using magnetic nanoparticles (MNP) [49].

### Therapeutic applications

#### Vaccine

As of now, the existing vaccines are inadequate to stop the spread of the COVID-19 disease due to the emergence of new variants. Fortunately, at least 16 additional vaccines have progressed to the clinical trial stage [50]. Furthermore, COVID-19 research is progressing to generate more vaccines to combat the disease. Currently, there are five types of vaccines available, which include live virus and inactivated vaccines; subunit vaccines; vector vaccines; and nucleic acid vaccines [51].

Subunit vaccines were developed using the SARS-CoV-2 protein, such as the S protein, which can trigger the immune system (Table 2). The spike protein was modified to produce stable spike protein in insect cells by substituting K986 and V987 in the S2 subunit with prolines (PP), resulting in S2 being cleaved into a minute fragment (Fig. 3) [16]. This fragment could be formed through proteolytic cleavage by the protease located within the S2 subunit [52]. Combining proline substitutions with another mutation that removes the cleavage site from the S protein results in a recombinant protein that provides maximum protection in vaccinated mice with minimum weight loss [16]. In addition, it was modified further using a mix of proline substitutions, the insertion of a C-terminal thrombin cleavage site, and the inclusion of an “AGAG” sequence at the furin cleavage site, resulting in S-2P, a recombinant protein [20]. The yield of the trimer-stabilised S-2P protein was greater than the wild-type spike protein in insect cells. S-2P is a promising vaccine candidate because it evokes high neutralising antibodies in monkeys and has significant reactivity with COVID-19 sera. It also had stronger immunogenicity at a lower inoculation dose. The S-2P protein was made soluble by replacing the transmembrane domain with a T4 foldon domain, resulting in a new recombinant spike protein called prefusion transmembrane-deleted spike (preS dTM) [53]. The preS dTM vaccine, which was adjuvanted with AS03 oil-in-water emulsion, generated a neutralising antibody response that protected nonhuman primates against a high dose of SARS-CoV-2 by giving protection in the upper and lower airways via a fast anamnestic response. Currently, this vaccine is progressing to a phase 3 clinical trial [54].

**Table 2:** Recombinant proteins produced in BEV for the development of COVID-19 vaccines

**Proteins**	**Modifications**	**Host Cells**	**Purification Method**	**Yield**	**Purity **	**Specific Applications**	**References**
Spike protein, S1 and receptor-binding domain (RBD)	Signal peptide, ectodomain, T4 foldon trimerization domain, thrombin cleavage site, spacer, ferritin, viral protein, and peptide tags added. Mutations introduced to the sequences.	Sf9/Sf21/High Five/ expresSF+/ BmN/silkworm larvae	Affinity or anion or gel filtration chromatography/ Ultrasonic crushing and ultracentrifugation/ Tangential flow filtration/Dialysis	0.19 - 30 mg/l	90 - 98%	Subunit vaccine	[16, 19, 55–59]
Spike protein, S1, receptor binding domain (RBD)	Codon-optimized and fusion with MERS-CoV S gene corresponding to the N-terminal domain (NTD) (RBD).	Sf9/293T	Not applicable	Not applicable	Not applicable	DNA Vaccine	[60]
Envelope, Membrane, Spike, S1 and influenza matrix protein 1 proteins	Signal peptides, prefusion stabilized ectodomain, T4 fibritin trimerization signal, peptide tags, linker, cleavage site, transmembrane and tail domain added. Codon optimization.	Sf9/ExpiSf9	Affinity chromatography/Sucrose gradient centrifugation	0.005 – 11 mg/mL	Not measured	VLP vaccine	[61–63]
Human angiotensin converting enzyme 2 (hACE2)	GP67 signal peptide sequence added at N terminus. Hexa-His tag added to the C terminus.	Sf9/High Five	Gel Filtration Chromatography	Not measured	Not measured	Surface plasmon resonance (SPR) assay	[64]

It was reported that the spike protein vaccine with Sepivac SWE™, a nanoemulsion oil (SWE) adjuvant, provided better protection to the challenged animals compared to the RBD vaccine using the same adjuvant [65]. Besides, other adjuvants, such as Advax-SM, were also found to be effective for protection against SARS-CoV-2 [66]. In other studies, it has been reported that the spike protein adjuvanted with gold nanoparticles induces a strong IgG response [67]. However, it fails to induce protective antibodies in the lungs.

A booster for the preS DTM vaccine derived from parental strain D614 or variant B.1.351 (Beta) has been formulated [68]. One dose of the vaccine formulated as monovalent D614 (parental) or B.1.351 (Beta) or bivalent (D614 + B.1.351) with AS03 adjuvant was found to significantly boost the existing neutralizing antibodies produced after previous vaccination in macaque (*Macaca mulatta*). Currently, these vaccines are at the clinical stage.

The spike protein is a trimer in its natural state, but when it is recombinantly generated, it mostly becomes monomeric [69]. To create trimeric recombinant spike proteins, the T4 foldon, an artificial trimerization domain derived from the bacteriophage T4 fibritin protein, was inserted into the S protein construct [55]. Another way to make spike protein trimers is to fuse *Helicobacter pylori* ferritin at the N-terminus [56]. Ferritin is a protein made up of 24 identical polypeptides that naturally produces nanoparticles [70]. The SGG linker binds ferritin to either the ectodomain (S1 and S2), the S1 or the RBD [56]. The recombinant virus was made in BmN cells while the large-scale nanoparticle vaccine was made in larvae. The ectodomain (ECD) trimer induced the highest level of antibodies.

The S protein’s immunogenicity is not restricted to its full component. Its subunits, such as S1 and RBD, can trigger the immune system on their own, but their antigenicity is lower than that of the entire unit [20, 60, 71]. The advantage of generating subunits rather than full-length spike proteins is that larger recombinant protein concentrations can be obtained (Table 2) [27]. Furthermore, when the plasmid vector was co-transfected with linearized baculovirus DNA defective in v-cath/chiAgenes, the protein amount was considerably greater [20]. In fact, it is the greatest SARS-CoV-2 protein yield ever produced using BEV for COVID-19 research. The S1 protein conjugated with fucoidan/trimethylchitosan nanoparticles (FUC-TMC NPs) and cytosine-phosphate-guanosine-oligodeoxynucleotides (CpG-ODNs) induces a broad spectrum neutralizing antibody response against SARS-CoV-2 variants [72]. Additionally, S1 protein was also coated with biopolymer particles (BP) to produce a vaccine that is stable at room temperature [57]. The binding of S1 protein with BP was mediated by the formation of an isopeptide bond between Spytagged S1 and SpyC displayed on the BP surface. This vaccine has been shown to provide protection for hamsters against SAR-CoV-2 infection.

RBD was used for the development of the COVID-19 vaccine using an adenovirus platform. The recombinant RBD was produced in an insect cell line before being displayed in the non-infectious adenovirus-inspired nanoparticle (ADDomer) [58]. Originally, the ADDomer platform was limited to simple antigens such as neutralizing epitopes from the Chikungunya virus. Its capability is further extended with the Spy Tag/Spy Catcher system, which enables the insertion of complex antigens such as RBD. The immunization of a mouse with the RBD vaccine elicited a significant anti-coronavirus humoral response, which was elevated further with the second vaccination. RBD was also fused to the rotavirus VP6-protein to create a fusion protein (FP) vaccine [59]. However, no RBD or S-specific antibodies were detected in the treated mice.

Apart from in vitro production, the S protein was also synthesized in vivo for the development of DNA vaccines by introducing DNA into the host using a baculovirus vector [60]. The CMV promoter was used to control the expression of the insert in the cells. The insertion of the envelope glycoprotein of human endogenous retrovirus (HERV) into the baculovirus genome improved the efficiency of vaccine gene delivery [73]. The recombinant baculovirus expressing SARS-CoV-2 full-length spike protein and the HERV gene (AcHERV-COVID19-S) was able to elicit neutralizing antibodies specific to SARS-CoV2 [60]. Additionally, virus titers were lower in the treated animals as well as fewer respiratory illnesses were observed in them. This result suggests that the vaccine could minimize SARS-CoV-2 infection.

Virus-like particle (VLP) is another type of COVID-19 vaccine synthesized using a baculovirus-insect cell platform that involves three techniques. In the first technique, the E, M, and S proteins were expressed in insect cells simultaneously using a triple expression plasmid [61]. Each component would be self-assembled in insect cells, forming a VLP. As for the second technique, the recombinant baculovirus expressing the full-length S, S1, or S2 proteins was co-transfected with another recombinant baculovirus expressing influenza matrix protein 1 (M1) to form VLP in the insect cells [62]. In the third technique, the S1 protein was coupled to the bacteriophage AP205 VLP nanoparticles [63]. This was done using the Spytag/Spycatcher platform. This was formulated as an adjuvanted vaccine.

The non-SARS-CoV-2 protein produced in BEV for a vaccine development study is human angiotensin converting enzyme 2 (hACE2) [64]. It was synthesized to investigate its binding to the SARS-CoV-2 RBD-dimer produced in mammalian cells. In this study, HACE2 had low affinity for the RBD, which could be due to inadequate glycosylation of human protein in insect cells [74]. Glycosylation in insect cells results in glycoproteins with simple oligo-mannose sugar chains, as contrasted to glycoproteins with complex sugar groups and terminal sialic acids in mammalian cells [75]. Transformed insect cells, like the SfSWT-1, can synthesize mammalian proteins with complex N-glycan [74].

To date, 10 vaccines that were created using different platforms have been approved for usage [76]. The only BEV-derived vaccine that obtained approval is NovaVax’s NVX-CoV2373 [50]. The NVX-CoV2373 vaccine exhibited 89.7% efficacy against SARS-CoV-2 infection and good efficacy against the B.1.1.7 variant after two doses [77]. There are three more BEV-derived vaccines that have reached the first or second clinical phase [50].

The biggest issue for producing successful vaccines is the emergence of new variants, as the presently licensed vaccines show decreasing neutralizations, particularly against the new Delta (B.1.617.2) and Omicron (B.1.1.529) variants [78, 79]. The two doses of inactivated vaccines followed by either a subunit, adenovirus-vectored vaccine, or mRNA vaccine, were found to be more efficient than homologous vaccines as a boost method [80]. Additionally, a vaccine that target the less mutated region such as M and E proteins might be able to neutralize any new variants. Side effects after vaccination is another shortcoming for the currently available vaccines. Indeed, it is one of the reasons for vaccine refusal. It was reported that the reactogenicity was dependent on the vaccine dosage where optimum dosage could help to minimize side effects such as headache, joint pain, diarrhea, and chilling [81]. Therefore, it is suggested that dosage need to be further optimized as to reduce the side effects, i.e., customizing the dosage according to the health status.

Cross-reactivity between different Adeno-associated viruses (AAV) serotypes has been reported to be 50% [61, 62]. BEV-derived vaccines offer an alternative to adenovirus-vectored vaccines, especially for people who develop Guillain-Barre Syndrome (GBS) after vaccination or who are undergoing gene therapy [82, 83]. Overall, the application of BEV for vaccine development was primarily focused on subunit vaccines, but fewer studies were done on DNA vaccines, where only one study has been identified. There are a lot of aspects that remain to be explored, such as the use of different eukaryotic promoters, which might affect the efficiency of the vaccine. In addition, BEV can express multiple proteins simultaneously, and this feature can be used for the development of novel vaccines that can trigger the production of multiple antibodies that can fight against different types of SARS-CoV2 variants. Moreover, this type of vaccine can cover a wide range of coronavirus diseases. The integration of nanotechnology into vaccine development has been proven to be successful in delivering new generation nucleic acid vaccines, particularly mRNA, into cells. Lipid nanoparticles (LNPs) have been used to protect mRNA from attack by ribonucleases and make it easier to get to the target site [14].

#### Protein and antibodies

The spike protein, which is essential for SARS-CoV or SARS-CoV-2 infection, has become a focus for therapeutic development (Table 3). The attachment of RBD to the cells is the first step in the SARS-CoV-2 pathogenesis. As a result, binding of antibodies to the RBD would limit the SARS-CoV-2 infection [84]. Several studies reported the production of S, S2, or RBD proteins in BEV to study the binding of the antibody in the serum of convalescent individuals [85–93]. It is also reported that optimization of several factors could lead to high yield antibody or antigen production in insect cell lines [94]. The inclusion of the Kozak sequence as well as the signal peptide of the mouse Ig heavy chain variable region in the vector increased the protein expression by more than 50%, with the optimum harvesting time being 96 h after transfection. Furthermore, it was also discovered that High Five cells grown in EX-CELL405 media yield the most protein. Additionally, the antibodies could be made into a lyophilized form without affecting their reactivity.

**Table 3:** Recombinant proteins produced in BEV for the development of COVID-19’s recombinant anti-bodies and therapeutic proteins

**Proteins**	**Modifications**	**Host Cells**	**Purification Method**	**Specific Applications**	**References**
Spike protein, S2, receptor binding domain, antibodies, and fusion proteins	Leader sequences, peptide tags signal peptide and restriction sites added.	High Five/silkworm larvae	Gel filtration and affinity chromatography	Surface plasmon resonance (SPR), ELISA	[87, 91, 92, 94–96]
Spike protein and antibodies	Signal peptides, pre-fusion stabi-lized ectodomain, T4 fibritin tri-merization signal, peptide tags, linker, cleavage site, linker, transmembrane and tail domain added. Mutations introduced to the sequences. Codon optimization.	High Five/ExpiSf9/silkworm larvae	Affinity chromatography	scFv, Fab, IgY and IgG antibody productions	[94, 95, 97, 98]
Spike protein and receptor binding domain	Signal peptide and peptide tag added. Biotinylation.	Sf9/High Five	Gel filtration, affinity, and size exclusion chromatography	Protein crystallizations	[85, 86, 89, 90, 93]
Receptor binding domain and angiotensin converting enzyme 2	Peptide tag added.	Sf9/High Five	Affinity chromatography	Cell sorting	[88, 99]
Spike protein and receptor binding domain	Biotinylation	Not mentioned	Not mentioned	B cell enrichment	[91]
Spike protein and receptor binding domain	Signal peptide and peptide tag added.	Sf9/High Five	Affinity and size exclusion chromatography	Binding assays	[93]

A monoclonal antibody against RBD, CR3022, has been produced using a silkworm-baculovirus expression vector system in three formats (scFv, Fab, and IgG) [95]. The affinity of the antibody towards S protein was equivalent to the one that was produced in the mammalian expression system. Additionally, the antibody production in silkworms gave a high yield as well as purity. The emergence of new SARS-CoV-2 variants could potentially reduce the efficiency of monoclonal antibodies (mAbs) and vaccines. Hence, a polyclonal anti-SARS-CoV-2 immunoglobulin was produced in transchromosomic (TC) bovines (Tc-hIgG-SARS-CoV-2) [97]. TC bovines are bovines that have been transformed with either human chromosome fragments, human artificial chromosomes, or mouse artificial chromosomes [100]. The advantage of using TC bovines to produce antibodies is that they can produce high amounts of human IgG in their serum [101].

To produce the antibodies against SARS-CoV-2, the TC bovines were given two doses of the DNA encoding the Wuhan-Hu-1 strain Spike gene, followed by three doses of S protein generated in insect cells [97]. SAB-185 antibodies were produced by purifying plasma from TC bovines. It was found that SARS-CoV-2 variants D614G, S477N, E484K, and N501Y could be neutralized by SAB-185. It is now in the clinical phase of testing. A therapeutic protein that targets SARS-CoV-2 spike protein has been developed and it is the tetravalent form of ACE2, which consists of four ACE2 extracellular domains coupled to the human immunoglobulin g1 Fc region [96]. This tetrameric ACE2 protein binds strongly to the RBD. Additionally, it also has more potential compared to monomeric (sACE2) and dimeric (ACE2-Fc) ACE2.

Most antibodies isolated from COVID-19 patients are specific to SARS-CoV-2. There are also some antibodies that can cross-neutralize other SARS-CoVs, including COVA1-16, H014, EY6A, S304, and CV38-142 [93, 102–105]. However, some of these antibodies have lower potency against the SARS-CoV-2 variant of concern [93]. It was found that a combination of the two cross-neutralizing antibodies such as CV38-142 and COVA1-16 showed enhanced neutralization towards the two SARS-CoV-2 variants of concern, B.1.1.7 and B.1.351. This synergistic effect occurred after the RBD bound to the COVA1-16 antibodies, where it was caught in the “up” state.

The S proteins produced in the BEV are also used for screening and isolation of nanobodies such as VHH. VHH nanobodies are heavy-chain only antibodies (hcAbs) generated by camelids or sharks that include a single variable domain in the antigen-binding segment of a naturally occurring IgG derivate [106, 107]. Nanobodies can be used to treat cancers, chronic disorders, as well as viral infections [107]. Alternatively, nanobodies can be produced using prokaryotic expression systems, which are highly scalable, rapid, and low-cost. A VHH library was created using bacteriophage and the specific nanobodies for SARS-CoV-2 S protein were screened, isolated, and tested against SARS-CoV-2 [106]. Using bacteriophage, a VHH library was generated, and specific nanobodies for SARS-CoV-2 S protein were screened, extracted, and tested against SARS-CoV-2 [96]. Using a bacterial display system, nanobodies can also be created without the need for bacteriophage [107]. *E. coli* bacteria in the bacterial display system contain intimin-Nanobody protein fusions that anchor in the outer membrane, exposing the functional nanobody to the extracellular environment for spike protein recognition, allowing the bacteria to adhere to spike protein-coated NHS-beads. The bacteria that express specific nanobodies travel all the way to the bottom of the Ficoll density gradient, leaving unbound bacteria in the upper fraction. The nanobodies were further characterized, cloned, and their capacity to neutralize SARS-CoV-2 was tested. The bacterial display technique allows nanobodies to be made with very little equipment and reagents.

To develop antibodies for COVID-19 disease, BEV is also being used to produce non-SARS-CoV-2 proteins such as ACE2 (Table 3). Two monoclonal antibodies (mAbs) specific for SARS-CoV-2 RBD generated by memory B cells from peripheral blood mononuclear cells have been discovered [99]. BEV-produced ACE2 for RBD binding was found to compete with CA1 and CB6 mABs. According to structure analysis, ACE2 and CB6 share numerous binding sites. CB6 binding induced steric hindrance, which was mediated by both the VH and VL domains of CB6, that prevented ACE2 from binding to the RBD. Currently, nine monoclonal antibodies are in clinical trials, with three of them, LY-CoV555 (Eli Lilly/AbCellera), REGN-COV2 (Regeneron), and CT-P59 (Celltrion), receiving Emergency Use Authorization (EUA) from the US Food and Drug Administration (FDA) [84]. In addition, ACE2 has also been developed as a therapeutic protein, where it has reached the clinical trial phase [108, 109]. Moreover, it has been found that substitutions of several ACE2 residues, such as S19, T27, and N330 with W19, W27, and Y330 could enhance their binding to SARS-CoV-2 S-RBD. This finding could become the basis for developing a new ACE2 therapeutic protein [110].

As a potential therapeutic for COVID-19 disease, SARS-CoV-2 S1 protein has been used for the production of egg yolk antibodies (IgY) [98]. The S1 protein produced in Sf9 insect cells was emulsified with Freund’s immune adjuvant and injected into hens. The IgY was extracted and tested against the SARS-CoV-2. The antibodies showed significant neutralizing potency against SARS-CoV-2.

Manufacturing antibodies against SARS-CoV-2 in vast quantities involves a high cost. The use of silkworm larvae to produce recombinant antibodies can reduce the production cost. In fact, it should be further investigated with larvae from other species, particularly those with fast growth and requiring minimum care. Additionally, the compatibility of larvae-derived antibodies with humans also needs to be investigated further.

#### Novel and repurposed drugs

**Fig. 4:**
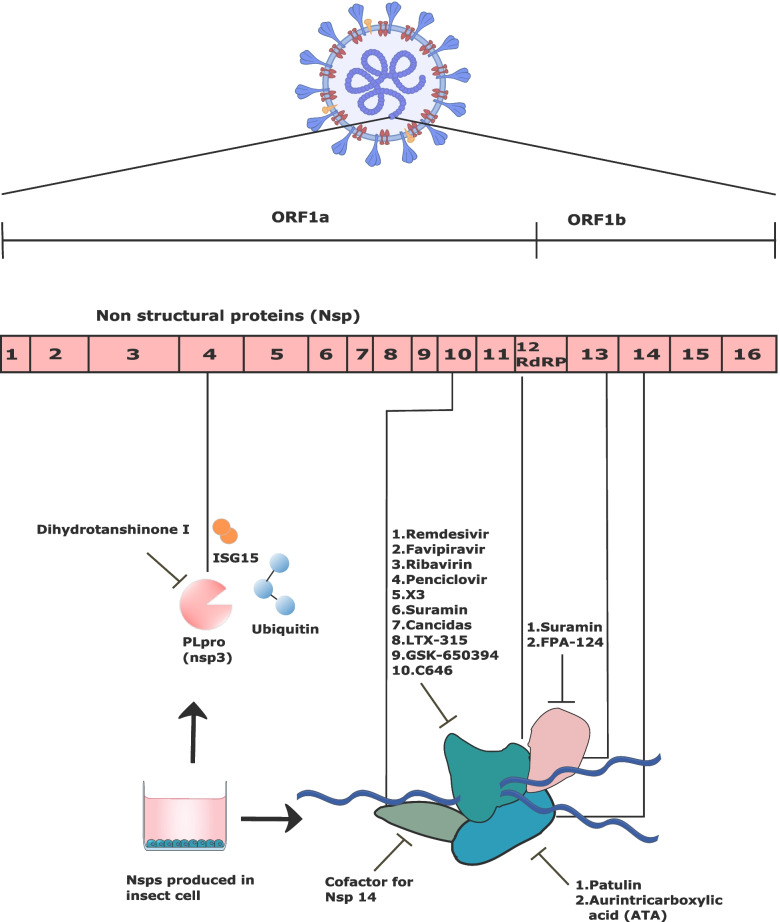
The NSPs produced in BEV and their inhibitors. There is a total of five SAR-COV-2 Nsp proteins produced in BEV and tested with their respective inhibitors. Nsp3 was inhibited by Dihydro-tanshinone I. Nsp 10 is a cofactor for Nsp14. Nsp12, which encodes for RdRP, was inhibited by 10 drugs (Remdesivir, Favipiravir, Penciclovir, X3, Suramin, Cancidas, LTX-315, GSK-650394, and Suramin). Suramin inhibited both Nsp12 and Nsp13. Nsp14 was inhibited by Patulin and ATA

The current situation of the COVID-19 pandemic demands immediate antiviral treatments to relieve the burden on the global healthcare system. The BEV platform has been used in research to find novel drugs as well as to study the antiviral activities of existing drugs against SARS-CoV2 (Table 4). Genome replication and translation of RNA viruses, including SARS-CoV-2, is carried out by RNA-dependent RNA polymerase (RdRP) [111]. RdRP is the polyprotein encoded by ORF-1a and ORF-1ab, which are located at the 5′-end of the SARS-CoV-2 genome (Fig. 4) [112].

**Table 4:** Recombinant proteins produced in BEV for the development of new drugs or repurposing the existing drugs for COVID-19

**Proteins**	**Modifications**	**Host Cells**	**Purification Method**	**Specific Applications**	**References**
Nsp12	Cleavage site and peptide tags added.	High Five	Size exclusion chromatography	In vitro polymerase inhibition assay	[113]
Nsp12, spike protein and receptor binding domain	Cleavage site changed. Trimerization domain, sequence motifs and peptide tags added. Codon optimization.	High Five	Affinity and size exclusion chromatography	Surface plasmon resonance (SPR)	[113–115]
RNA-dependent RNA polymerase (RdRp) complexes (nsp5, nsp7, nsp8, nsp10, nsp12 and nsp14)	Peptide tags, cleavage site, linker and sequence motifs added. Codon optimization.	Sf9	Affinity and size exclusion chromatography	RNA synthesis assay	[116–122]
Nsp13	Peptide tags added. Codon optimization.	Sf9	Affinity chromatography	Helicase assay	[123]
S1	No modification.	Sf9/*Tnao*38	Affinity chromatography	Platelet adhesion assay, In vitro thrombus formation and Flow cytometry measurement of fibrinogen binding.	[124]
Nsp9	Peptide tags added. Codon optimization.	Sf9	Affinity chromatography	Gel based nsp9 cleavage assay	[125]
papain-like protease (PLpro)	Peptide tags added. Codon optimization.	Sf9	Not mentioned	Protease assay	[126]
Spike protein and receptor binding domain	A trimerization domain added. Cleavage site added.	High Five	Not mentioned	ELISA	[115]

The two ORFs encode precursor polyproteins for 16 nonstructural proteins (nsp1 to nsp16) which form the replicase–transcriptase complex (RTC) that consists of multiple enzymes, including papain-like protease (nsp3), chymotrypsin-like main protease (3CL protease, nsp5), primase complex (nsp7 and nsp8), RdRP (nsp12), helicase (nsp13), and exoribonuclease (nsp14) [127]. Because RdRp has no host cell homolog, its inhibitors would have no effect on proteins in human cells. This has resulted in RdRP becoming the target for nucleotide analogue drugs such as Remdesivir (RDV) and Favipiravir [116, 127]. The RdRP complex (nsp5-7-8-12) polyproteins produced in insect cells will be cleaved by the nsp5 protease into an active binary nsp8/12 RdRp complex. Alternatively, nsp12, nsp7, and nsp8 can be produced by generating separate baculoviruses for each protein [117]. In addition, nsp proteins were also produced in different expression systems. For example, nsp12 was produced in BEV while its cofactors nsp7 and nsp8 were expressed in the *E. coli* expression system [113]. Further investigation found that the expression of the RdRP complex was highly increased with the addition of the His6-3xFlag tag on nsp12 (Sf nsp12-HF) and a neutral 6 amino acid (Gly-Gly-Ser) 2-linker between nsp7 and nsp8 [118].

Nucleotide analogue drugs are produced in their triphosphate form to act as substrates for RdRP, which results in termination of RNA synthesis [116]. The termination of RNA synthesis by Remdesivir triphosphate form (RDV-TP) does not occur immediately. Its incorporation at position ‘i’ commonly results in delayed chain termination between positions i+3 and i+5 after a few nucleotide incorporation events. The cryo-electron microscopy (cryo-EM) images of RdRP in complex with the template, primer double-stranded RNA (dsRNA), and favipiravir ribonucleoside triphosphate (favipiravir-RTP) revealed that favipiravir, which was present at the active site of RdRP, stacks onto the 3′ nucleotide of the primer strand by forming a noncanonical base pair with the cytosine in the template strand [56]. Favipiravir, like other nucleotide analogues such as ribavirin and penciclovir, binds to + 1 sites at the RdRP complex, but due to the drug’s nonproductive conformation in the polymerase active site, it is only weakly incorporated into the RNA primer strand [117]. The 3′OH of the P-1 nucleotide in the template strand is not orientated for nucleophilic attack on the β-phosphate of favipiravir-RTP [119].

Favipiravir, ribavirin, and penciclovir were found to be less effective than remdesivir [117]. Remdesivir, despite this, is difficult to synthesize, administer, and can even cause hepatotoxicity. As a result, it was further modified to obtain oral delivery as well as a uniform distribution of the nucleoside and nucleotide metabolites throughout the body, particularly in organs that are particularly susceptible to SARS-CoV-2, such as the lungs. The insertion of a deuterium atom at the C5 position of the base moiety, as well as esterification with isobutyric acid, are examples of such changes. The X3 molecule showed a 5-fold increased replication inhibition compared to the original remdesivir.

Suramin is another potential drug for treating COVID-19, which is at least 20-fold more potent than remdesivir against SARS-CoV-2 [120]. The inhibition mechanism of suramin is the same as remdesivir but it occupies a different base position at RdRP. Additionally, suramin also interferes with multiple steps of coronavirus infection and replication, which include interruption of host-cell interaction, cellular uptake, as well as inhibition of viral helicase activities [123]. There are several limitations which hinder the application of suramin for the treatment of COVID-19, such as its high negative charge that restricts its entry into cells, potential off-target effects on other cellular enzymes and exposure to endocytosis [120, 123]. The uptake rate of suramin by the cells can be improved by liposomal delivery [123]. Caspofungin acetate (Cancidas) and the oncolytic peptide LTX-315 are another two potential drugs for treating COVID-19 [113]. Other potential compounds include R406 (fostamatinib) and ibrutinib [124]. Several novel compounds that have the potential for treating COVID-19 have been discovered. Such drugs are GSK-650394 and C646 [118]. These compounds were found to inhibit SARS-CoV-2 RdRp activity at non-cytotoxic concentrations.

Molnupiravir is another oral drug that can serve as a substrate for RNA polymerases. It can be taken orally and intracellularly metabolized to its triphosphate form (NHC-TP) [121]. However, using RdRP synthesized in the BEV, it was found that the natural nucleotide, Cytidine triphosphate (CTP), is 30-fold preferable to NHC-TP. Besides synthetic compounds, there are also natural alkaloids that were found to bind to SARS-CoV-2 RdRP, such as emetine and cephaeline [114]. Emetine is an alkaloid that comes from the root of the ipecac plant, *Carapichea ipecacuanha*. Cephaeline is a desmethyl version of emetine.

There are a total of at least nine enzymatic activities in SARS-CoV-2 which are potential for drug targets [125]. Those enzymes and their cofactors that have been produced using BEV are nsp3, nsp5, nsp10, nsp13, and nsp14 (Table 4) [116, 122, 123, 126]. Nsp3 contains a papain-like protease (PLpro) which generates viral non-structural proteins from a polyprotein precursor [126]. Recombinant nsp3 produced in insect cells was less active compared to the nsp3 produced in bacteria. The PLpro activity was inhibited by dihydrotanshinone I, a derivative of tanshinones, which resulted in inhibition of SARS-CoV-2 proliferation at an EC_50_ of 8 μM. Additionally, dihydrotanshinone I did not exhibit much cytotoxicity, even at high concentrations. Further investigation showed that it has no synergistic effect with remdesivir.

Nsp14 is an exoribonuclease/methyltransferase whose cofactor is nsp10. It reduces the host innate antiviral immune response by cleaving viral-associated double-stranded RNAs and by regulating viral genome recombination [122]. It became the target for developing a new antivirus for COVID-19. As of nsp12/7/8, nsp14 and nsp10 were more active when they were fused together with a short linker.

Nsp13 encodes a viral helicase and hence plays an essential role in viral replication and proliferation where it unwinds DNA or RNA in an NTP-dependent manner with a 5′ to 3′ polarity [123]. As opposed to nsp3, nsp13 produced in insect cells was more active than nsp13 produced in bacteria [126]. Nsp13 might be more active in its glycosylated form, which results from post-translational modifications in insect cells. Suramin and FPA-124 were found to be potential nsp13 inhibitors with lower IC_50_ in vitro and lower anti-viral EC_50_ in cell-based assays as compared to the previously reported compounds, myricetin and SSYA10-001 [123]. The nsps produced by BEV and its inhibitors are illustrated in Fig. 4.

It has been reported that lipids play a role in the suppression of viral infection [128]. The essential free fatty acid linoleic acid (LA) is a lipid that binds to the SARS-CoV-2 spike protein at RBD [115]. The attachment is provided by an arginine (Arg408) and a glutamine (Gln409) from the adjacent RBD. LA reduced the binding of RBD to ACE2 by stabilizing the closed conformation of the SARS-CO-V2 spike protein. The S protein is only accessible in an open state, that is, when the RBD is in an up position [129]. Further investigation found that there is a synergy between LA and remdesivir. The remdesivir dosage is reduced with the addition of LA.

Nanoceria is a rare earth nanoparticle that possesses promising anti-inflammatory properties by inhibition of NFκB and MAPK pathways, which could halt the progression of systemic inflammatory complications in COVID-19 patients [130]. Bilirubin, decorin, and silver nanoparticles are other nanomedicines that act in almost similar ways to nanoceria [131–133].

As of now, only four drugs are approved for treating COVID-19 [134]. Paxlovid and Molnupiravir are orally available, while Sotrovimab and Remdesivir are administered through intravenous infusion. There are numerous antiviral drugs on the market, and BEV-derived protein can be used to simulate the antiviral effects of these drugs on SARS-CoV-2. Moreover, the antiviral activities of natural alkaloids or plant-derived compounds have been less investigated.

## Conclusions

The baculovirus expression vector technology was not only relevant until today, but also proved to be highly beneficial in dealing with the current COVID-19 pandemic. However, given its complexity in comparison to other expression systems such as bacteria and yeast, there is always room for improvement. To summarize, the spike protein is the most cloned SARS-COV-2 protein, with Ni-NTA affinity chromatography being the most common purification method and the His tag being the most utilized protein tag in BEV for the COVID-19 study. Currently, the only BEV-derived vaccine approved for usage is subunit vaccines. Therefore, more studies are required to obtain approval for the other types of vaccines.

The use of nanoparticles led to high sensitivity and fast diagnosis as well as improved drug delivery for COVID-19. However, nanoparticles are expensive to produce and require strong infrastructure. At present, affordable conventional drugs are effective enough to treat COVID-19. Therefore, expensive nanomedicine is not a wise choice for the time being. Still, nanotechnology is expected to get cheaper and more widely available over time.

The emergence of the new variants has made the existing detection and treatment less efficient. As a result, more studies are required to generate new knowledge about the COVID-19 disease in order to develop new innovations that can cater to all types of SARS-CoV-2 variants. In view of this, the continuous improvement of the BEV can play an important role. Scalability and flexibility are among the crucial elements for such improvements. The availability of a highly customizable platform with a low manufacturing cost is very useful, especially for dealing with future pandemics. It is predicted that for BEV, the use of insect larvae for large-scale and low-cost recombinant protein production will be expanded. Lastly, the baculovirus vector is expected to be reinvented continuously to obtain a high yield of the recombinant protein with minimum input.

## Supplementary Information

**Additional file 1: Table S1.** The specific queries for PubMed, ScienceDirect, Scopus and Google Scholar databases.

## Data Availability

Not applicable.

## Abbreviations

ACE2: Angiotensin converting enzyme 2
ACE2-Fc: Dimeric ACE2
AcHERV: *Autographa californica* HERV
AAV: Adeno-associated viruses
BEV: Baculovirus expression vector system
CMV: Cauliflower mosaic virus
COVID-19: Coronavirus disease
CTD: C-terminal domain
DNA: Deoxyribonucleic acid
dsRNA: Double-stranded ribonucleic acid
ECD: Ectodomain
E: Envelope protein
EUA: Emergency use authorization
Favipiravir-RTP: Favipiravir ribonucleoside triphosphate
GBS: Guillain-Barre Syndrome
hcAbs: Heavy-chain only Abs
hACE: Human ACE2
HERV: Human endogenous retrovirus
M: Membrane protein
MCF: Microcapillary film
mRNA: Messenger RNA
IgA: Immunoglobulin A
IgG: Immunoglobulin G
IgM: Immunoglobulin M
LA: Linoleic acid
MMLVRT: Moloney murine leukemia virus reverse transcriptase
mAbs: Monoclonal antibodies
N: Nucleocapsid proteins
Nsps: Non-structural proteins
NTD: N-terminal domain
ORF: Open reading frame
PLpro: Papain-like protease
preS dTM: Prefusion transmembrane-deleted spike
RBD: Receptor binding domain
RDV-TP: Remdesivir triphosphate
RdRP: RNA-dependent RNA polymerase
S: Spike protein
sACE2: Monomeric ACE2
SARS-CoV-2: Severe acute respiratory syndrome coronavirus-2
TC: Transchromosomic
U.S FDA: United States United States Food and Drug Administration
VHH: Nanobodies
