# Supplementary material for: Application of Baculovirus Expression Vector system (BEV) for COVID-19 diagnostics and therapeutics: a review
Source: J Genet Eng Biotechnol. 2022 Jul 6;20:98. doi: 10.1186/s43141-022-00368-7 (PMC9259773; doi:10.1186/s43141-022-00368-7)
Supplement: Supplementary file 1 — Additional file 1: Table S1. The specific queries for PubMed, ScienceDirect, Scopus and Google Scholar databases. [file 43141_2022_368_MOESM1_ESM.pdf]

Table S1. The specific queries for PubMed, ScienceDirect, Scopus and Google Scholar databases.

**PubMed Search Strategy**	
**Query**	**Results**
baculovirus expression AND (sars-cov-2 OR 2019nCoV OR severe acute respiratory syndrome coronavirus 2)	17
**ScienceDirect Search Strategy**	
**Query**	**Results**
baculovirus AND expression (sar-cov-2 OR 2019nCoV OR severe acute respiratory syndrome coronavirus 2)	472
**Scopus Search Strategy**	
**Query**	**Results**
ALL (baculovirus AND expression AND (sars-cov-2 OR 2019ncov OR 'severe AND acute AND respiratory AND syndrome AND coronavirus AND 2'))	758
**Google Scholar Search Strategy**	
**Query**	**Results**
"baculovirus expression" AND ("sars-cov-2" OR "2019nCoV" OR "severe acute respiratory syndrome coronavirus 2"))	520
